# Alumni survey of Masters of Public Health (MPH) training at the Hanoi School of Public Health

**DOI:** 10.1186/1478-4491-5-24

**Published:** 2007-10-19

**Authors:** Linh Cu Le, Quyen Tu Bui, Ha Thanh Nguyen, Arie Rotem

**Affiliations:** 1 Department of Graduate Education, Hanoi School of Public Health, Vietnam; 2 Department of Biostatistics, Hanoi School of Public Health, Vietnam; 3 Department of Graduate Education, Hanoi School of Public Health, Vietnam; 4 Advisor to Hanoi School of Public Health on Educational Development, Vietnam

## Abstract

**Background:**

1) To elicit the opinions of the Public Health alumni of the MPH program; 2) To assess the applicability of the knowledge and skills acquired; 3) To identify the frequency of the public health competencies that the alumni performed.

**Methods:**

We requested 187 graduates to complete a self-administered questionnaire and conducted in-depth interviews with 8 alumni as well as a focus group discussion with 14 alumni.

**Results:**

In total 79.1% (148) of the MPH graduates completed and returned the questionnaire. Most alumni (91%) agreed that the MPH curriculum corresponded with the working requirements of public health professionals; and nearly all were satisfied with what they have learnt (96%). Most respondents said that the MPH program enabled them to develop relevant professional skills (95%) and that they were satisfied with the curriculum (90%). Notably fewer respondents (73%) felt that the MPH program structure was balanced and well designed. Most alumni (64.3%) were satisfied with Hanoi School of Public Health (HSPH) full-time lecturers; but even more (83%) were satisfied with visiting lecturers. The most commonly selected of the 34 pre-identified public health competencies were: applying computer skills (66.4%), planning and managing health programs (47.9%), communicating with the community and/or mobilizing the community to participate in health care (43.2%). Overall, the MPH alumni felt that HSPH emphasized research methods at the expense of some management and operational competencies. The most important challenges at work identified by the alumni were insufficient skills in: data analysis, decision making, inter-sectoral cooperation development, English language and training.

**Conclusion:**

The training program should be reviewed and revised to meet the needs of its graduates who enter diverse situations and positions. English language skills were identified as top priority for further emphasis. The training program should comply with a more advanced accreditation system and standards.

## Introduction

Hanoi School of Public Health (HSPH) is a leading academic institution in Vietnam which provides training, conducts research, and informs the policies of the Ministry of Health in the broad area of public health. Among these tasks, training is considered to be the most important. In 1995, on direction of the Ministry of Health, the school started developing the Master of Public Health (MPH) program. Since 1996, it has been cooperating with many international experts in applying a pioneering approach in Vietnam entitled "Public health school without walls – PHSWOW", as an active member of the international PHSWOW network. Unlike traditional training programs in which field training and practice is poorly emphasized, the School's MPH program lasts two years, of which one is field-based. By 2006, seven cohorts had completed the program with a total of 187 MPH graduates. The initial program intake was about 20–25 students per year. Recently, the school's annual intake ranges from 40 to 50 MPH students including students from the Lao People's Democratic Republic and Cambodia.

During this period there has been one comprehensive evaluation of the training program carried out by an expert team supported by the Rockefeller fund at the end of 2000 [1]. The course manuals, academic staff and infrastructure were regarded as appropriate for MPH training and the field-based study in the second year was considered very practical. Qualitative interviews with six students of the school also reflected appreciation of the school's efforts to support the students. The students felt more confident and more knowledgeable about public health as a result of the training received.

In developing the training programs, HSPH referred to many professional documents and conducted several studies on core public health functions. A study conducted by HSPH in collaboration with the World Health Organization (WHO) identified the key public health functions that public health professionals at different levels are required to perform [2]. The research findings were used as a basis for developing training programs for MPH ensuring that the students' capacity fits the needs of their work. However, to date, it has not been verified that students were adequately prepared to perform key public health functions in practice.

Recently, HSPH has developed an educational development master-plan for the next 10 years. The focal point in the initial five year phase is the evaluation of the adequacy of training and initiation of quality management of all training programs. The current survey contributes to this evaluation by reviewing alumni perceptions of the MPH program at HSPH as well as their progress and career path. This is the first detailed review of MPH training programs in Vietnam. It offers an assessment of the alumni's acquired competencies in relation to a framework of the essential functions of public health.

A cross-sectional survey was conducted to: 1) Elicit opinions of the public health alumni on the MPH program; 2) Assess the relevance and adequacy of the knowledge and skills acquired during the program; 3) Identify the relative importance of a range of public health competencies performed by alumni in their present work situation.

## Methods

### Cross-sectional quantitative survey

The self-administered questionnaire was sent out to all MPH alumni of HSPH. Graduates who were studying or working abroad were contacted by e-mail. The research announcement and contents were presented on the website of the Department of Graduate training HSPH for reference. In total 148 graduates out of the total of 187 responded to the survey and returned a completed questionnaire (see [Additional file 1]).

### Qualitative research

An in-depth interview was conducted with five MPH alumni who were working as lecturers at HSPH. These interviews served as a pre-test leading to revision of the questionnaire. Group discussions with 14 representatives from the seven MPH cohorts were conducted in the format of a workshop. Initially, we prepared a list of 21 alumni (including three members from each cohort) based on their current positions, gender and post/functions. Given the qualitative nature of this study and logistic constraints, we did not aim to select the participants on a random basis, but aimed to achieve thorough representation of the student body. Due to personal reasons, some of the alumni invited were not able to attend. The final 14 who attended were relatively representative of the cohorts: approximately two per cohorts, except for cohorts six and seven, who have just graduated, from which we had just one participant per cohort (more were invited, but were unable to attend). This was compensated by the fact that we did more in-depth interview with these cohorts, as described below. With regard to position and current job, the 14 alumni interviewed were quite representative. Twelve of them were working in public/government sector (including Ministry of Health offices, training institutions, research institutes, provincial health offices, preventive medicine center, etc.) and two were working for non governmental organizations (NGOs)/international organizations. Among these 14 alumni, 10 are from Hanoi, three are from other provinces in the northern part of Vietnam and one is from a central province. Within this mini workshop, structured group discussion and nominal group techniques were used to obtain information. This approach was adapted from other study methodologies [3,4].

In addition to the mini workshop, eight in-depth interviews were conducted with MPH alumni (mainly from North Central and Central Coast provinces). Among these, four were males and four were females. We selected two alumni from the first cohort (one is now a senior lecturer in a medical school, one is a district hospital director), one from cohort four who is now working for an international organization, one alumni from cohort five who is now a teacher at a secondary medical school, one from cohort six who is working within the communication sector at provincial level, and three from cohort seven (one at secondary medical school, two at provincial health offices). The researchers travelled to these provinces to meet and interview four alumni, four others were interviewed during their visit to Hanoi.

The data was checked and entered using a data based programmed with Microsoft.NET tool, and managed in Microsoft Access 2003. Descriptive and bivariate analysis methods were applied, followed by multivariate techniques (including factor analysis.). For the results of in-depth interviews thematic analysis was applied; the data from group discussions and interviews were analyzed based on the list of discussed topics and their prioritized order. Data analysis was carried out with SPSS, version 12.0.

## Results

The majority of the alumni are now working in the northern part of the country (87%), mainly Hanoi (47.3% of the total sample). There are a few international students who have returned to their countries (two in Cambodia and four in the Lao People's Democratic Republic), and one alumnus is living in the United States of America. The overall response rate was 79.1% (148/187). Males accounted for 56.1% and females accounted for 43.9% of the sample. The current mean age of the alumni is 40.8 (41.5 in males and 39.8 in females). More than 57% of the alumni are in the age group 36–45. The mean age of MPH students at the beginning of their program is 35.3. No significant difference was evident in the mean age at enrolment when we compared across cohorts: The mean age at enrolment of the MPH1 is: 35.8, MPH2: 34.9, MPH3: 33.6, MPH4: 35.3, MPH5: 34.8, MPH6: 38.0, MPH7: 34.5. With regard to current alumni employment: 43.2% are working at provincial public organizations (versus 45.3% prior to MPH training), 38.5% are working at national/ministry government organization (versus 30.4% prior to MPH training), 9.5% working at district level (versus 15.5% prior to MPH training), 4.7% working for international agencies/NGOs (versus only 1.4% prior to MPH training), about 4% working for private organization/company and others (versus 5.4% prior to MPH training). The majority of these alumni are working in preventive medicine public health sector (58.9%), 15% are working in curative medicine sector and the remainder are working in other health and non-health fields.

In general the alumni reported promotion to higher ranks after the training. Data showed that prior to MPH training, 59.3% of the alumni were at regular staff status (versus 43.9% currently), 24.8% were head of a department of an institution/organization (versus 31.1% currently), none of them were head of department at ministry level (versus 0.7% currently), and 8.3% of them were head of an institution/organization (versus 18.9% currently). It is difficult to claim that all promotion relates to their MPH training, but the qualitative interviews suggested that training made a major contribution to their career prospects.

The most frequent tasks that the alumni have to perform are: training (74.8%), preparing plans (72.1%), and conducting research (57.8%). The tasks that they perform least frequently are: evaluation and management of para-clinical services (18.4%) and providing clinical services (19.7%). Out of the alumni, 93% said that the tasks and functions they perform correspond well with what they learnt in the MPH program.

### 1. Identification of opinions of the public health alumni on the MPH program

**Figure 1:**
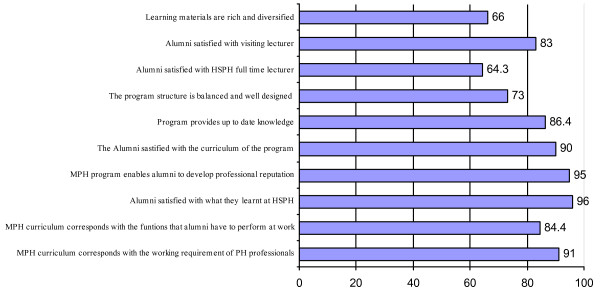
General opinions of the Public health alumni on the MPH program (percent agree).

In general, alumni were satisfied with the MPH program and the vast majority of them recognized that the program helped to improve their professional reputation, and that the design of the program corresponded with requirements of the practice reality (Figure 1).

"*I usually encourage others to apply to study at HSPH because the study program is appropriate and very relevant to our work.... The training management at the School is very strict, they check our progress on a regular base and this is good for us. The field training supervision is very demanding, but it helps, it enables students to reach higher levels of learning. Studying here is very demanding, it's really a burden compared to other universities, but the quality is definitely much better. [FGD, female, MPH4]*

Table 1 indicates that some of the respondents thought that there was too much emphasis on epidemiology and research methodology (7.4% each). But these subjects were also ranked highest in the importance level of routine work (84.4% and 87.7%, respectively), just after basic computer application skills (92.5%). In contrast, the subjects that the alumni comment as "not emphasized enough" are: qualitative method (48% of the alumni), English (49.7%) and data analysis (46.6%).

**Table 1:** Alumni's perspective on specific subjects/topics (percent, n = 142)

	***Subjects/topics***	***Emphasis was given to this area in the MPH course (%)***			***The importance in everyday work (%)***		
		
		*Not enough emphasis*	*Appropriate emphasis*	*Too much emphasis*	*Not important*	*Some importance*	*Important*
1	Basic computer skills	35.4	63.3	1.4	0.0	7.5	92.5
2	Basic epidemiology	6.1	86.5	7.4	2.7	12.9	84.4
3	Basic statistics	29.9	60.0	4.1	4.1	29.3	66.7
4	Data analysis skills	46.6	50.7	2.7	2.1	22.6	75.3
5	Demography	5.5	89.0	5.5	6.8	43.8	49.3
6	Disease prevention	24.3	73.6	1.4	6.8	31.3	61.9
7	English	49.7	49.7	0.7	2.1	28.8	69.2
8	Environmental health	21.6	75.7	2.7	4.8	41.5	53.7
9	Health economics	33.3	62.6	4.1	12.3	34.9	52.7
10	Health education and health promotion	20.3	77.7	2.0	1.4	17.8	80.8
11	Health policy	28.6	65.3	6.1	3.4	28.1	68.5
12	Health system management	10.9	89.1	0.0	1.4	15.8	82.9
13	Maternal and child health care	24.7	70.5	3.4	14.5	38.6	46.9
14	Occupational health	23.6	73.0	2.0	13.6	49.0	37.4
15	Pedagogy	20.9	77.0	1.4	8.2	26.5	65.3
16	Qualitative methodology	48.0	48.0	2.7	2.7	30.6	66.7
17	Rehabilitation	30.6	66.0	0.7	19.4	56.9	23.6
18	Research methodology	13.5	79.1	7.4	0.7	11.6	87.7
19	Seminar	19.6	75.7	4.7	0.7	22.4	76.9

The subjects which alumni suggested as least important in their work were rehabilitation, occupational health, maternal child health care and demography. This is not surprising since these are more specialized areas than general management of public health programs and many of the graduates are not assigned to work in these areas.

The alumni's comments in the open-ended section of the questionnaire are mainly related to the following issues: 1) the need for more data analysis training, scientific report writing, presentation/seminar skills for MPH students; 2) the need for more flexibility in relation to field work exercises and thesis topics, allowing students to do thesis at their work places; 3) the need for optional subjects and the modification of some "less important" subjects; 4) the need to develop specialized MPH tracks; 5) the need to improve the capacity of lecturers at HSPH, noting that many members of staff are still young and inexperienced.

The graduates in general indicated that they were proud to be HSPH alumni. They reported high confidence in performing public health activities and confidence in their ability to do research in particular. Overall they felt that they received a high quality of training with up-to-date knowledge with emphasis on practical skills. Key competencies and skills most frequently used and mentioned by the alumni were research skills, data analysis using statistical software, planning and evaluation, team work, communication skills and health education, problem solving, etc.

The respondents indicated that the overall structure of the MPH program was appropriate. However, they identified several areas that need to be modified. HSPH, in their view, should definitely move towards an accreditation and quality management system so that the students can accumulate their credits and transfer to other institutions. The MPH program should be clearly divided into core courses and optional (elective courses) to help develop "specialized MPH graduates". They identified emerging issues that could be developed into new optional courses such as: injury epidemiology, non-communicable diseases, and qualitative research methods especially particularly rapid appraisal (PRA).

"*The training program should include both core (mandatory) courses and elective courses. For example, because I conduct many training activities I need to learn more about training methodology, on the other hand, courses such as occupational health is not used in my job, which is really wasteful." [FGD, female, MPH5]*

"*Advanced universities in the world have mandatory courses and elective courses in their training programs. Our School should follow them." [FGD, female, MPH2]*

The overall impression is that full time faculty members were well trained and applied active teaching methods. Part-time and guest lecturers were not regarded as high quality (though this view is not consistent with the quantitative data findings).

The English training at HSPH was considered "rather good" but very far from perfection. Respondents indicated that there was too much theory, and not enough practice or communicative lessons. The content of the course seems to be too "simple" and general and not sufficiently focused on the content and context of language used in Public Health.

Computer skills and data analysis appear to be a strength of MPH alumni. They highly appreciated those training courses at HSPH and showed significant confidence in relation to this area. However, they still expressed the need to study more data analysis skills and techniques.

Public speaking was considered a very important skill for MPH alumni. Their experiences showed that this is usually a weak point of public health professionals as a whole. Therefore, more attention should be given to this specific skill in MPH. The alumni also highly recommend HSPH to continue the weekly seminar for the students to prepare, present and talk in front of their classmates and teachers.

The alumni highly valued the field training component of the program. They all concluded that this is a must and that this experience helps them to considerably build up their own research capacity. The advantages of this component include: practicability, real-life experiences, hands-on teaching and learning and rigorous supervision during field work. In addition, many of the alumni suggested that HSPH should allow for the option of doing the field work in groups, instead of as individuals (first exercise of four months) with the second period of six months (field work exercise number two) leading to a thesis, done individually.

"*Not only skills to do research but also skills to deal with problems that the research discovers, which means providing interventions, are necessary. The school is strong in training in research methodology. Thus its alumni can do research very well and confidently. However, they lack many practical skills, which cause difficulties for the alumni in designing and implementing intervention programs. In the second year, which is field-based, the focus is only identifying problems. Meanwhile, the next steps to solve the problems such as identifying resources, making intervention plan, and supervising the implementation of intervention are not emphasized*.

When I return to work in the Department of Preventive Medicine, Ministry of Health (MOH), we mainly implement programs, not research. When I am asked to identify problems and write a plan based on Logframe, I cannot do that. In the MPH program, there are only one or two lessons on Logframe. It is necessary to modify the training curriculum, creating balance between research and project management skills which means besides focusing on research, courses in the first year should be re-designed so that they are not in the status of being redundant in one aspect while still inadequate in the others." [FGD, male, MPH5]

### 2. MPH alumni's perspective on public health skills and competencies

These 34 competencies were presented in four major groups: Public health management, Training, Research/evaluation, and Leadership competencies (Table 2). The detailed list of 34 competencies with frequency of how often the alumni practice and their confidence when performing these competencies is presented in [Additional file 2].

**Table 2:** The percentage of frequency of competencies performed by the MPH alumni

	***3 most frequently performed***			***3 least frequently performed***		
	***1***	***2***	***3***	***1***	***2***	***3***

***Public health management***	Plan and manage health programs (47.9%)	Monitor health problems and epidemics in the community (37%)	Develop indicators and instruments to monitor community health (35.2%)	Evaluate and develop public health regulations (17.9%)	Describe the health system structure and the drivers of health system change (25.3%)	Consult in making public health policies and plans (25.3%)
***Training***	Provide training in public health (37.7%)	Develop health-related capacity building plans and strategies (37.6%)	Monitor and evaluate a training program (33.8%)	Evaluate the health human resource in terms of quality, quantity and need (29%)		
***Research/evaluation***	Apply computer skills successfully in your work (66.4%)	Collect health information in a community (41.1%)	Assess and analyze the health situation of a community (35.6%)	Apply the procedures of the Ethics committee in biomedical studies (15.3%)	Apply qualitative methods in public health practice (20.5%)	Use English effectively in your work (22.1%)
***Leadership***	Communicate with the community, mobilizing the community to participate in health care activities (43.2%)	Facilitate group work effective (39.5%)	Use analytical, critical thinking and problem-solving skills to make decisions effectively (39.55%)	Work effectively within culturally diverse groups and settings (26.7%)	Lobby leaders for solving community health problems (28.8%)	Create multi sectoral cooperation to solve community health problems effectively (29.3%)

The data shows that competency number five (plan and manage health programs) is the most frequently performed by the alumni among the 10 public health management competencies (47.9% of them applied this competency very frequently), followed by monitor health problems and epidemics in the community (37%), and develop indicators and instruments to monitor community health (35.2%). The least frequently performed competency is evaluate and develop public health regulations (17.9%). Interestingly, the competency plan and manage health programs is the one that the vast majority of the alumni feel most confident about (96.9%). The next one is design health promoting interventions for the community (90.1%), followed by monitor health problems and epidemics in the community (88.5%).

With regard to the training competencies, it was found that about 38% of the alumni have provided training in public health very frequently and also about 38% of the alumni have to develop health-related capacity building plans and strategies as a frequent task. The level of confidence of the alumni in relation to providing training in public health and developing health-related capacity building plans and strategies is quite high (91.7% and 88%, respectively).

The next group composed 12 competencies/skills to conduct research and evaluation in public health. With regard to these competencies, apply computer skills successfully was the one that they most frequently performed (66.4%), followed by collect health information in a community (41.1%). It is noteworthy that only 36.5% of the alumni feel confident about using English effectively, and this is the lowest level of confidence among all 34 public health competencies. Thus, although almost 70% of the alumni confirmed that English is very important in their current work (66.2% of them replied that they sometimes need to use English, 22% need to use English very frequently), only 36.5% of the respondents feel confident about their English proficiency. Logistic regression analysis was conducted to explore the variables that relate to their 'confidence' in using English. After controlling for age, MPH cohorts, positions, levels of work (national, provincial, NGOs, etc.), working areas (health, non-health, etc.), and gender, the only factor found to be statistically related to the likelihood of not being confident in using English was the perception whether English was important or not (p < 0.001). Specifically, those who expressed their opinion that English was not (or minimally) important were actually 3.1 times more likely to be less confident in English, compared with their alumni counterparts who considered that English was important.

Regarding the fourth group of competency, leadership in public health, the competency that they perform most frequently is communicating with the community, mobilizing the community to participate in health care activities (43.2%). This is also the competency with which the highest percentage of alumni feels confident in (95.6%). At the opposite end, the one they performed least frequently and felt least confident about was work effectively within culturally diverse groups and settings.

### 3. MPH alumni's perspective on their current challenges at work

During the group discussions, alumni were requested to share their views and experiences concerning the current work they were undertaking. Importantly, we asked them to share the challenges that they were facing at their current work. Group nominal technique was used to explore this issue. The alumni admitted that they have to deal with several issues at work.

We used group nominal technique to sort out the most important challenges through the scoring process (Table 3). The top three challenges were: the lack of data analysis skills, lack of decision making skills and the difficulty in developing intersectoral cooperation (the same score of 17). The next concern was English proficiency and the fifth concern was training competency.

**Table 3:** The challenges of the MPH alumni in work settings, classified by competency groups

**Public health management competencies**	**Teaching/training competencies**	**Research/evaluation competencies**	**Leadership competencies**
- Lack of skills to evaluate health programs at lower level	- Lack of skill to encourage student to study - Lack of skill to train subordinates and lower-level health staff - Poor self-study competency	- Lack of injury, health economic research development skill - Lack of data analysis skill - Poor Public Health English capacity - Lack of skill to search for information in the Internet	- Lack of policy advocacy skill - Lack of group conflict solving skill - Lack of skill to persuade and explain to colleagues and leader - Lack of skills to work in culturally diverse settings - Lack of skill to persuade and explain to clients - Lack of decision making skill - Difficulty in creating intersectoral cooperation

## Discussion

The current distribution of the MPH alumni by geographic regions indicates that the MPH program at HSPH has provided public health education primarily for professionals from the northern part of Vietnam, with a lot of the alumni now working in Hanoi.

The comparison before and after MPH training shows a clear trend that the MPH graduates have gained certain promotions after their graduation. The proportion of alumni who worked for low level in health care sector (district and below) decreased while the proportion of those working for national level and international organizations increased after the MPH training. The percentage of alumni who have become head of the departments at institutions and head/deputy head of institutions has increased.

The overall structure of the MPH program appears to be satisfactory with one year of school-based and one year of practical/field-based education. However, there is a need to develop the curriculum geared towards a flexible approach, just like accredited MPH programs in developed countries, which allow students to study core (compulsory) and optional (elective) subjects. Suggestions concerning optional courses included: community-based rehabilitation, nutrition, disease prevention, occupational health and maternal child health care. There were some other themes that could be provided as elective subjects, such as project/program development, injury prevention, HIV/AIDS prevention, and non-communicable diseases. Alumni highlighted the importance for HSPH to move towards an accreditation and quality management system so that they could gain recognition for their studies by other institutions. In addition, alumni identified several areas they would like to study in more depth, including advanced data analysis skills, qualitative methods, and professional English. With regard to English proficiency, the alumni highly recommended that HSPH should provide more skills on using English as a tool to access public health scientific documents/references in English over the Internet, to summarize papers and reports in English.

The need to track the MPH program into (at least) two directions is clear. Most of the students indicated that research is not the only issue, and in most cases it is not the main task that they perform in routine work. Therefore, it is obvious that the research-component of the MPH program should be maintained, but to complement that, more practical/management-based components are required for the majority of MPH students. This could be the very first step of the development of more specialized MPH programs. Therefore, HSPH needs to immediately consider different tracking options. At least two tracks are needed: research-based (strong focus on research methodology) and program/practical-based (strong focus on project development and management).

In addition to existing academic subjects, some other skills are also very important. They include: scientific report writing, presentation/seminar skills, how to access information over the Internet, public speaking, and self-study competency, etc.

More flexibility in terms of field work exercise requirement and thesis topics is an emerging issue. The students would appreciate HSPH to allow them: 1) to implement the first field work exercise in groups rather than individuals; 2) to conduct thesis research at their work places, analyse secondary data, etc.

There is the need to improve the capacity of lecturers at HSPH, as many of the staff are still young and inexperienced. This would also link to the capacity building process at HSPH as well as the staff's academic requirement and staff evaluation process at HSPH. This also includes the criteria to screen, select and invite guest/visiting lecturers.

Some other academic skills newly identified as critical such as scientific report writing, presentation/seminar skills, information search over the Internet, public speaking, and self-study competency, have raised the issue of new subjects and coursework needed. Therefore, these topics should be discussed and assigned to specific academic departments and groups to develop and introduce to the students. In conjunction with this process, library and information services should also be reviewed and improved.

## Conclusion

For the long-term strategy, HSPH should move forward an action plan of training quality assurance and quality control. This should initially address the following issues: capacity building for junior and inexperienced teaching staffs (with strong focus on pedagogy), staff performance evaluation regulations and processes, student's feedback and evaluation of teaching quality (at the end of each subject and at the end of the whole program) and learning materials reviews and modification/adaptation

The competency to plan and manage health programs is the most frequently performed by the alumni among the 10 public health management competencies. The next competencies are: monitor health problems and epidemics in the community and develop indicators and instruments to monitor community health. These findings suggest that the management and administrative workload is very crucial for MPH alumni. This finding is consistent with studies conducted in Australia where generic skills associated with report writing, project coordination and all aspects of planning and management also featured very highly [5,6].

As discussed above, the English capacity is of major concern for the MPH graduates. This issue is confirmed by the fact that few of the alumni said that they feel confident about using English effectively, and this is the lowest level of confidence among all 34 public health competencies. Knowledge of English is regarded as important for further professional development and thus for promotion and career prospects.

As part of a long term strategy for educational development the HSPH should adopt a "comprehensive approach" using problem-based learning (or scenario-based learning) approaches, so that the "domains" of competencies can be better addressed.

## Competing interests

The author(s) declare that they have no competing interests.

## Authors' contributions

LCL was responsible for the overall design and conduct of this study, the development of the questionnaire, the collection and interpretation of data, drafting the article, and editing of the final version.

QTB was responsible for the development of the questionnaire and supported the data collection, the quantitative data analysis.

HTN was responsible for the coordination of this study, the development of the questionnaire, the data collection, qualitative data analysis.

AR contributed to the conceptualization of the study, the development of the questionnaire, support the interpretation of the findings and editing of the final version.

## Supplementary Material

Additional file 1 Quantitative self-administered questionnaire. The questionnaires used in this survey. Click here for file

Additional file 2 Public health skills and competencies. These tables provide more details about the Public Health skills and competencies that performed by the alumni. Click here for file
