# Supplementary material for: Alumni survey of Masters of Public Health (MPH) training at the Hanoi School of Public Health
Source: Hum Resour Health. 2007 Oct 19;5:24. doi: 10.1186/1478-4491-5-24 (PMC2186353; doi:10.1186/1478-4491-5-24)
Supplement: Additional file 1 — Quantitative self-administered questionnaire. The questionnaires used in this survey. [file 1478-4491-5-24-S1.doc]

## Additional file 1 – Quantitative self-administered questionnaire

# HANOI SCHOOL OF PUBLIC HEALTH

**MASTER OF PUBLIC HEALTH PROGRAM EVALUATION QUESTIONNAIRE**

First of all, Hanoi School of Public Health would like to send you the warmest greetings.

Over the past ten years of operating and developing the MPH program, Hanoi School of Public Health has made a significant contribution to building capacity for the health sector. In order to further improve the quality of the training program, we conduct this survey to explore your opinions on the program and the possibility of applying the knowledge and skills that you learned from the program in your work. At the same time, we would like through this survey to promote the development of the HSPH alumni network in order to strengthen linkages among alumni of different MPH courses as well as to enhance your long-term collaboration with us.

We would like to assure you that the information you provide will serve research purposes and help improve our training program and will not be used for other purpose Your participation in the survey is entirely voluntary. We highly appreciate your contribution and are really grateful for your collaboration .While completing the questionnaire, should you have any questions please contact our staff:

Nguyen Thanh Ha

Department of Graduate Training

Telephone: 04 266 2335

Email: nth1@hsph.edu.vn

Upon completion of this questionnaire, please put it in the prepaid stamped envelop that we provided and send it to us by mail as soon as you can. Thank you very much!

**Instruction for completing the questionnaire:**

- **For information that needs to be filled in the blank, please write neatly.**
- **For multiple choice questions: circle one or more numbers that correspond to your choice(s) (see example below)**
- **When you have already circled a number but wish to change your decision or if the number does not correspond to your choice, please cross the number you have circled then circle the number that corresponds to your final choice**
- **Please use pen or ball pen, only black or blue ink should be used. Pls. do not use pencil. Try to avoid erasing. If you really need to erase, please use correction pen with white liquid then continue completing the questionnaire.**

**Example: For the question below, if your answer is "unmarried", you should circle number 3. If you make a mistake by circling number 1, then you should fully cross it and then circle number 3 as illustrated below**

|  | What is your current marital status? | Living with husband/wife 1  Divorced/separated/widowed 2  Unmarried 3 |  |
| --- | --- | --- | --- |

**SECTION 1. BACKGROUND INFORMATION**

Please read the questions carefully. Circle the number(s) that correspond(s) to your choice(s) or fill in the blanks with appropriate information

| **No** | **Question** | **Answer** | | | **Note** | |
| --- | --- | --- | --- | --- | --- | --- |
|  | Year of birth | ………………………………………………… | | |  | |
|  | Sex | Male 1  Female 2 | | |  | |
|  | What is your current marital status? | Living with husband/wife 1  Divorced/separated/widowed 2  Unmarried 3 | | |  | |
|  | What MPH course did you follow? | Course 1 (1997-1999)  Course 2 (1998-2000)  Course 3 (1999-2001)  Course 4 (2000- 2002)  Course 5 (2001-2003)  Course 6 (2002- 2004)  Course 7 (2003- 2005) | 1  2  3  4  5  6  7 | |  | |
|  | What degrees or qualifications did you hold **before starting your MPH program?**  *(List* ***at most*** *2 degrees or qualifications of university or college level upward. Example of answer: General doctor, Thai Binh Medical University, 1985)* | | | | | |
|  | Degrees/qualifications | ___________________________ | |  | |  |
|  | Training institution | ___________________________ | |  | |  |
|  | Year of graduation | ⬜ ⬜ ⬜ ⬜ | |  | |  |
|  | Degrees/qualifications | ___________________________ | |  | |  |
|  | Training institution | ___________________________ | |  | |  |
|  | Year of graduation | ⬜ ⬜ ⬜ ⬜ | |  | |  |

**SECTION 2. OCCUPATION BEFORE AND AFTER ATTENDING THE MPH PROGRAM**

| **No** | **Question** | | | **Answer** | | **Note** |
| --- | --- | --- | --- | --- | --- | --- |
| **Before starting the MPH program** | | | | | | |
|  | What kind of organization did you work for? | At home, not having any official job  National government organization/ ministry-governed organization  Provincial government organization  District government organization  Commune government organization  International organization/ NGO  Private organization/company  Other (please specify) ……………… | | | 1  2    3  4  5  6  7  8 | **Skip to 2.6** |
|  | What is your organization's working area | Non-health field  Preventive medicine/Public health  Curative medicine  Other (Please specify)…………........... | | | 1  2  3  4 |  |
|  | What was your position before attending the MPH course | Staff  Head of a department of an institution/organization  Head of a department of a ministry  Head/deputy head of an institution/organization  Other (please specify)…….................. | | | 1  2  3  4  5 |  |
|  | In that past job, what were the functions you performed?  (*Please circle as many as apply )* | Managing programs/projects  Consulting on policies  Making plans  Training  Conducting health programs  Evaluating and managing curative services  Evaluating and managing para-clinical services  Working on health communication and education and health promotion  Conducting studies  Doing administrative work (personnel development, financing, etc)  Providing clinical services  Other (please specify) ….………..….. | | | 1  2  3  4  5  6  7  8  9  10  11  12 |  |
|  | Among these functions, please tell us **one main** function on which you spent most of your time  ***(fill in the box the number that corresponds to your choice from previous question)*** | | | | ⬜ |  |
| **After completing the MPH program** | | | | | | |
|  | After completing the MPH course, did you change your job? | | Yes, I moved to another institution  No, I had no change in working institution  I still work for the same institution but at another position | | 1  2  3 | **Skip to 2.10** |
|  | If you have moved to another organization or position, how many times have you changed since your graduation? | | Once  Twice  Three times  More than three times | | 1  2  3  4 |  |
|  | What kind of organization are you working for currently? | | At home, not having any official job  National government organization/ ministry-governed organization  Provincial government organization  District government organization  Commune government organization  International organization/ NGO  Private organization/company  Other (please specify) …………… | | 1  2    3  4  5  6  7  8 |  |
|  | What is your organization's working area? | | Non-medical  Preventive medicine/Public Health  Curative medicine  Other (Please specify) ....................... | | 1  2  3  4 |  |
|  | What is your current position? | | Staff  Head of a department  Head of a department of a ministry  Head/deputy head of an institution/organization  Other (please specify) ….................. | | 1  2  3  4  5 |  |
|  | In your job, what are the **functions** that you perform?  (*Please circle as many as apply )* | | Managing programs/projects  Consulting on policies  Making plans  Training  Conducting health programs  Evaluating and managing curative services  Evaluating and managing paraclinical services  Working on health communication and education and health promotion  Researching  Doing administrative work (personnel development, financing, etc)  Providing clinical services  Other (please specify) ………..…… | | 1  2  3  4  5  6  7  8  9  10  11  12 |  |
|  | Among these functions, please tell us **one** **main** function that you spend most of your time  ***(fill in the box the number that corresponds to your choice)*** | | | | ⬜ |  |
|  | Do you think that function corresponds with what you learned from the MPH program? | | Yes  No  Don't know | | 1  2  3 |  |
|  | Do you intend to look for another job? | | Yes  No  I haven't known yet | | 1  2  3 | Skip to 2.16  Skip to 2.16 |
|  | If yes, what field do you intend to work in? | | Managing programs/projects  Consulting on policies  Making plans  Training  Conducting health programs  Evaluating, managing curative services  Evaluating, managing paraclinical services  Working on health communication and education and health promotion  Conducting studies  Administrative work (personnel development, financing, etc)  Other (please specify) ………..….. | | 1  2  3  4  5  6  7  8  9  1  10  11 |  |

|  | Do you intend to study further to improve you qualification? (Following long-term training programs such as PhD, master, bachelor programs...) | Yes  No  I haven't thought of it yet | 1  2  3 | Skip to 3.1  Skip to 3.1 |
| --- | --- | --- | --- | --- |
|  | If yes, which program will it be? | Bachelor  Master  PhD  Post – doctor) | 1  2  3  4 |  |
|  | What field will be the major focus of the program you intend to follow? | Public health/preventive medicine  Curative medicine  Non-health field  Other (please specify) …...……..….. | 1  2  3  4 |  |

SECTION 3. REASONS FOR FOLLOWING THE MPH PROGRAM

|  | What **reasons** encouraged you to follow an MPH program? ***(You may have more than one choice)*** | To find a job  To increase chances of promotion  To increase your income  To improve your professional competence  To find a new job  To change your career path  To expand knowledge and skills  Your employer suggested it  You had a scholarship  Dissatisfaction with previous professional practice  Other (please specify…………..….. | 1  2  3  4  5  6  7  8  9  10  11 |  |
| --- | --- | --- | --- | --- |
|  | Among those reasons, which is the main one?  ***(choose one number that corresponds to your choice and fill in the box)*** | | ⬜ |  |
|  | For **what reasons** did you choose the MPH program of Hanoi School of Public Health?  ***(Please circle as many as apply))*** | The school's prestige  High quality of the training program  Qualified academic staff  Good learning environment (adequate facilities, teaching equipment, etc.)  Convenient and comfortable accommodation  Appropriate training duration  Having support in terms of scholarship, learning equipment (laptop, materials, etc.)  My friends introduced the program to me  Other (please specify) ………….... | 1  2  3  4  5  6  7  8  9 |  |
|  | Among those reasons, which is the **main one**?  ***(choose one number that corresponds to your choice and fill it in the box)*** | | ⬜ |  |
|  | Have you ever introduced the program that you followed to anyone else? | Yes  No | 1  2 | **Skip to section 4** |
|  | Do you intend to introduce the program that you have followed to others in the future? | Yes  No  No answer | 1  2  3 |  |

**SECTION 4: PUBLIC HEALTH KNOWLEDGE AND SKIILS /COMPETENCIES**

In this section, we use scales to assess some key aspects related to the topic areas and subjects that you learned in the MPH program at Hanoi School of Public Health.

Please read carefully the contents of the subjects/topics listed below, and then **circle one** appropriate number in each column. ***Don’t forget to check the scales we provide***

***A- KNOWLEDGE***

| ***#*** | ***Title of subject /topic area*** | ***How much emphasis was given to this area in the MPH course*** | ***How important is this area in your everyday work*** |
| --- | --- | --- | --- |
|  |  | *0-Not offered*  *1- Not enough emphasis*  *2- Appreciated emphasis*  *3- Too much emphasis* | *1.Not important*  *2- of some importance*  *3- important* |
| 4.1 | ***Maternal and child health care*** describes maternal physiological changes before, during and after delivery. It also discusses important issues of maternal and child nutrition and care. | 0 1 2 3 | 1 2 3 |
| 4.2 | ***Health policy*** analyses current health policies, with comparison and analysis based on lessons from other countries. It also helps learners to develop appropriate evidence-based polices. | 0 1 2 3 | 1 2 3 |
| 4.3 | ***Demography*** introduces basic demographic indicators (CBR, CMR, etc.), demographic models, demographic equations, etc... | 0 1 2 3 | 1 2 3 |
| 4.4 | ***Basic epidemiology*** introduces fundamental epidemiological principles, measures and types of epidemiological studies, etc. | 0 1 2 3 | 1 2 3 |
| 4.5 | ***Health education and health promotion*** provides communication methods, skills and teamwork skills as well as strategies to promote community health, including use of leaflets, loudspeakers. It also analyses the relation between behavioral science and health. | 0 1 2 3 | 1 2 3 |
| 4.6 | ***Health economics*** provides basic concepts of health economics, calculations of health expenditures. It also introduces Vietnam health insurance system. | 0 1 2 3 | 1 2 3 |
| 4.7 | ***Research methodology*** provides steps to conduct a biomedical research, guidelines to write a feasible research proposal, guidelines to write report of research findings. | 0 1 2 3 | 1 2 3 |
| 4.8 | ***English*** skills enables learners to use English in communication and in professional work | 0 1 2 3 | 1 2 3 |
| 4.9 | ***Data analysis skills***: enable learners to use popular data-analysis software programs such as EPIINFO, SPSS, etc. in managing, analyzing and interpreting survey data. | 0 1 2 3 | 1 2 3 |
| 4.10 | ***Disease prevention***  provides principles of disease prevention and control | 0 1 2 3 | 1 2 3 |
| 4.11 | ***Rehabilitation*** describes disabilities. It also introduces possibility and methods of community based rehabilitation for each type of disability | 0 1 2 3 | 1 2 3 |
| 4.12 | ***Pedagogic methodology*** provides fundamental principles of adult teaching. It also introduces teaching methods and skills applied in medical field. | 0 1 2 3 | 1 2 3 |
| 4.13 | ***Health system management*** enables learners to identify health problems, to draw a problem tree or fishbone diagram to identify root causes of the problem, to write research objectives, to make intervention plans. | 0 1 2 3 | 1 2 3 |
| 4.14 | ***Environmental health*** describes current environmental health problems. It introduces methods to protect the environment sustain ably. It also discusses the impact of the environment on human health. | 0 1 2 3 | 1 2 3 |
| 4.15 | ***Basic statistics***: It introduces statistical calculations and statistical tests. It also discusses interpretation of research data | 0 1 2 3 | 1 2 3 |
| 4.16 | ***Basic computer skills:*** enables learners to use office software programs such as Microsoft word, Microsoft excel, Microsoft PowerPoint as well as take advantage of the Internet | 0 1 2 3 | 1 2 3 |
| 4.17 | ***Occupational health*** discusses occupational diseases and how to prevent them | 0 1 2 3 | 1 2 3 |
| 4.18 | ***Qualitative methodology*** enables learners to design and conduct a study merely using qualitative methods or a study using both qualitative and quantitative methods. It also provides learners with basic qualitative data-collection skills such as in-depth interview, focus group discussion, etc. | 0 1 2 3 | 1 2 3 |
| 4.19 | ***Seminar***: In each seminar, learners present a health problem to an audience, discussing and analyzing the problem. | 0 1 2 3 | 1 2 3 |

***B-COMPETENCIES***

***Please indicate how often you use each of the following competencies in your current work and rate how confident you feel in performing each competency***

| ***Title of competencies*** | ***How frequently are you using this competency in current work?*** | ***How confident do you feel in performing this competency*** |
| --- | --- | --- |
|  | *1-Not at all*  *2- Sometime*  *3- Very frequently* | *1-Not confident at all*  *2-Fairly confident*  *3- Confident*  *4- Very confident* |
| ***A. Public health management competencies*** | | |
| 1. Being able to describe the health system structure and the drivers of health system change | 1 2 3 | 1 2 3 4 |
| 1. Being able to identify the scope, role and functions of public health in relation to the health system, other sectors and to civil societies. | 1 2 3 | 1 2 3 4 |
| 1. Being able to evaluate the health care system in order to make policies relating to health care services | 1 2 3 | 1 2 3 4 |
| 1. Being able to monitor health problems and epidemics in the community | 1 2 3 | 1 2 3 4 |
| 1. Being able to plan and manage health programs | 1 2 3 | 1 2 3 4 |
| 1. Being able to manage health programs and services: increasing the accessibility of health services and decreasing inequity in health care | 1 2 3 | 1 2 3 4 |
| 1. Being able to develop indicators and instruments to monitor community health. | 1 2 3 | 1 2 3 4 |
| 1. Being able to design a health promoting interventions for the community | 1 2 3 | 1 2 3 4 |
| 1. Being able to consult in making public health policies and plans | 1 2 3 | 1 2 3 4 |
| 1. Being able to evaluate and develop public health regulations | 1 2 3 | 1 2 3 4 |
| ***B. Training competencies*** | | |
| 1. Being able to evaluate the health human resource in terms of quality, quantity and need | 1 2 3 | 1 2 3 4 |
| 1. Being able to develop health-related capacity building plans and strategies | 1 2 3 | 1 2 3 4 |
| 1. Being able to provide training in public health | 1 2 3 | 1 2 3 4 |
| 1. Being able to monitor and evaluate a training program |  |  |
| ***C. Research/evaluation competencies*** | | |
| 1. Being able to collect health information in a community | 1 2 3 | 1 2 3 4 |
| 1. Being able to assess and analyze the health situation of a community | 1 2 3 | 1 2 3 4 |
| 1. Being able to obtain adequate resources for a research | 1 2 3 | 1 2 3 4 |
| 1. Being able to organize a community- based study | 1 2 3 | 1 2 3 4 |
| 1. Being able to develop a public health research proposal | 1 2 3 | 1 2 3 4 |
| 1. Being able to collect research data in the field | 1 2 3 | 1 2 3 4 |
| 1. Being able to make use of information technology for effective data analysis and communication | 1 2 3 | 1 2 3 4 |
| 1. Being able to apply qualitative/quantitative methods in public health practice. | 1 2 3 | 1 2 3 4 |
| 1. Being able to write a research report | 1 2 3 | 1 2 3 4 |
| 1. Being able to apply the procedures of the Ethic committee in biomedical studies | 1 2 3 | 1 2 3 4 |
| 1. Being able to apply computer skills successfully in your work | 1 2 3 | 1 2 3 4 |
| 1. Being able to use English effectively in your work | 1 2 3 | 1 2 3 4 |
| ***D. Leadership competencies*** | | |
| 1. Being able to communicate with the community, mobilizing the community to participate in health care activities | 1 2 3 | 1 2 3 4 |
| 1. Being able to conduct communication activities on the community’s health problems | 1 2 3 | 1 2 3 4 |
| 1. Being able to demonstrate effective written and oral communication with the community in various contexts. | 1 2 3 | 1 2 3 4 |
| 1. Being able to lobby leaders for solving community health problems | 1 2 3 | 1 2 3 4 |
| 1. Being able to create multi-sectoral cooperation to solve community health problems effectively | 1 2 3 | 1 2 3 4 |
| 1. Being able to facilitate group work effectively | 1 2 3 | 1 2 3 4 |
| 1. Being able to use analytical, critical thinking, and problem-solving skills to make decisions effectively. | 1 2 3 | 1 2 3 4 |
| 1. Being able to work effectively within culturally diverse groups and settings. | 1 2 3 | 1 2 3 4 |

**SECTION 5: GENERAL EVALUATION OF TRAINING ACTIVITIES**

**5.1. *As you have followed the MPH program at HSPH, please give your general opinions on the training program.***

*(Please tick****Ö*** *the appropriate box)*

**1 2 3 4 5**

**Absolutely Disagree Don't know Agree Absolutely**

| **disagree agree** |  |  |  |  |  |
| --- | --- | --- | --- | --- | --- |
| **5.1. *Your general opinions on the MPH program of HSPH*** | **1** | **2** | **3** | **4** | **5** |
| 1. The design of the program corresponds with the requirements in the work of public health professionals. |  |  |  |  |  |
| 1. The quantity of subjects in the MPH program is appropriate |  |  |  |  |  |
| 1. The overall structure of the curriculum is well designed and balanced |  |  |  |  |  |
| 1. The MPH program provides up-to-date knowledge |  |  |  |  |  |
| 1. The MPH program corresponds with your functions that you perform in your current work |  |  |  |  |  |

***5.2.*** Facilities and support at HSPH

|  | | **1** | | **2** | | **3** | **4** | **5** |
| --- | --- | --- | --- | --- | --- | --- | --- | --- |
| 1. Accommodation at the school’s dormitory is comfortable and convenient | |  | |  | |  |  |  |
| 1. Teaching equipment is good | |  | |  | |  |  |  |
| 1. Classroom condition is good | |  | |  | |  |  |  |
| 1. Learning materials are rich and diversified | |  | |  | |  |  |  |
| **5.3. *Other opinions on HSPH*** | **1** | | **2** | | **3** | | **4** | **5** |
| 1. In general, you are satisfied with the school's full-time lecturers |  | |  | |  | |  |  |
| 1. In general, you are satisfied with the school's visiting lecturers |  | |  | |  | |  |  |
| 1. In general, you are satisfied with the curriculum of the MPH program |  | |  | |  | |  |  |
| 1. In general, you are satisfied with what you learned at the school |  | |  | |  | |  |  |
| 1. The supporting staff provided you with adequate support and services during the time you study at HSPH |  | |  | |  | |  |  |
| 1. Staffs at the school's field sites provided adequate support to you during the field work exercises. |  | |  | |  | |  |  |
| 1. The MPH program helps to develop your professional reputation |  | |  | |  | |  |  |
| 1. The MPH program enables you to increase your income |  | |  | |  | |  |  |

**5.4. *In the MPH program that you followed, were there any subjects/topics which you consider important to your work in particular and to the work of others in the field of public health that were not offered?***

Yes  No  don't know/no answer

If yes, please list those subjects/topics that need to be included in the MPH program:

……………………………………………………………………………………………………………………………………………………………………………………………………**5.5. *Besides the information you have provided above, if you have any suggestions for the improvement of the MPH program of HSPH, please write them below:***

………………………………………………………………………………………………

**5.6. *If you want to keep in touch and further cooperate with the school, please suggest how you wish the school to support you and what you wish to contribute to the school:***

………………………………………………………………………………………………

***If you don't mind please fill in some up-to-date information so that we can contact you in the future***

Your full name: ...........................................................................................................

Office address :...................................................................................................................

Office telephone: .......................................Fax: ..........................Cell phone:.........................

Email: ........................................................................................................................ ........

Home address:.................................................................................................................

Telephone: ........................................ Fax: .........................................................................

***Thank you very much for your collaboration!***

***We wish you success in your work and life. We hope that you will cooperate with us in HSPH's activities. We also hope that you will provide us with more information and initiate activities to strengthen the HSPH alumni network. Please keep in touch with us and visit our website regularly to have up-to-date information.***

***Please contact us at:***

***Department of Graduate Education -Hanoi School of Public Health***

***138 Giang Vo, Hanoi, Vietnam***

***(Room 2.1, 2^nd^ floor, Building A)***

***Telephone:   84 4 266 2335***

***Fax:      84 4 266 2385***

***Website: www.hsph.edu.vn/gt***
