# Supplementary material for: Alumni survey of Masters of Public Health (MPH) training at the Hanoi School of Public Health
Source: Hum Resour Health. 2007 Oct 19;5:24. doi: 10.1186/1478-4491-5-24 (PMC2186353; doi:10.1186/1478-4491-5-24)
Supplement: Additional file 2 — Public health skills and competencies. These tables provide more details about the Public Health skills and competencies that performed by the alumni. [file 1478-4491-5-24-S2.doc]

## Additional file 2 - Public health skills and competencies

**Table A - Public health management competencies performed by the MPH alumni**

*(each competency is presented with the note of sample size replied to the 2 questions of how frequently they use and how confident they feel when performing)*

|  | ***Skills/ competencies*** | ***How frequently do alumni use this competency in current work (%)*** | | | ***How confident do alumni feel in performing this competency (%)*** | | |
| --- | --- | --- | --- | --- | --- | --- | --- |
|  |  | *Not at all* | *Sometime* | *Very frequently* | *Not confident at all* | *Some confident* | *Confident* |
| ***Public health management competencies*** | | | | | | | |
| **1** | **Describe the health system structure and the drivers of health system change** | **20.5** | **54.1** | **25.3** | **0.9** | **20.0** | **79.1** |
| 2 | Identify the scope, role and functions of public health in relation to the health system, other sectors and to civil societies (n=144/120) | 17.1 | 54.1 | 28.8 | 0.0 | 17.4 | 82.6 |
| **3** | **Evaluate the health care system in order to make policies relating to health care services (n=144/120)** | **17.1** | **52.1** | **30.8** | **0.8** | **27.3** | **71.9** |
| 4 | Monitor health problems and epidemics in the community (n=144/119) | 17.1 | 45.9 | 37.0 | 0.8 | 10.7 | 88.5 |
| **5** | **Plan and manage health programs (n=144/128)** | **11.0** | **41.1** | **47.9** | **0.0** | **3.1** | **96.9** |
| 6 | Manage health programs and services: increasing the accessibility of health services and decreasing inequity in health care (n=143/109) | 24.1 | 44.8 | 31.0 | 0.0 | 15.1 | 84.9 |
| **7** | **Develop indicators and instruments to monitor community health (n=143/123)** | **14.5** | **50.3** | **35.2** | **0.0** | **16.0** | **84.0** |
| 8 | Design a health promoting interventions for the community (n=143/119) | 15.9 | 58.6 | 25.5 | 0.0 | 9.9 | 90.1 |
| **9** | **Consult in making public health policies and plans (n=144/121)** | **16.4** | **58.2** | **25.3** | **2.5** | **14.8** | **82.7** |
| 10 | Evaluate and develop public health regulations (n=143/107) | 24.8 | 57.2 | 17.9 | 2.8 | 29.6 | 67.6 |

**Table B - Training competencies performed by the MPH alumni**

|  | ***Skills/ competencies*** | ***How frequently do alumni use this competency in current work (%)*** | | | ***How confident do alumni feel in performing this competency (%)*** | | |
| --- | --- | --- | --- | --- | --- | --- | --- |
|  |  | *Not at all* | *Sometime* | *Very frequently* | *Not confident at all* | *Some confident* | *Confident* |
| ***Training competencies*** | | | | | | | |
| **11** | **Evaluate the health human resource in terms of quality, quantity and need (n=143/123)** | **14.5** | **56.6** | **29.0** | **0.0** | **9.7** | **90.3** |
| 12 | Develop health-related capacity building plans and strategies (n=143/124) | 13.8 | 49.7 | 37.6 | 0.0 | 12.0 | 88.0 |
| **13** | **Provide training in public health (n=144/131)** | **9.6** | **52.7** | **37.7** | **0.8** | **7.5** | **91.7** |
| 14 | Monitor and evaluate a training program (n=143/124) | 12.4 | 53.8 | 33.8 | 0.8 | 8.7 | 90.5 |

**Table C - Research/evaluation competencies performed by the MPH alumni**

|  | ***Skills/ competencies*** | ***How frequently do alumni use this competency in current work (%)*** | | | ***How confident do alumni feel in performing this competency (%)*** | | |
| --- | --- | --- | --- | --- | --- | --- | --- |
|  |  | *Not at all* | *Sometime* | *Very frequently* | *Not confident at all* | *Some confident* | *Confident* |
| ***Research/evaluation competencies*** | | | | | | | |
| **15** | **Collect health information in a community (144/139)** | **3.4** | **55.5** | **41.1** | **0.0** | **2.8** | **97.2** |
| 16 | Assess and analyze the health situation of a community (n=144/133) | 7.5 | 56.8 | 35.6 | 0.0 | 7.4 | 92.6 |
| **17** | **Obtain adequate resources for a research (n=145/132)** | **7.5** | **61.2** | **31.3** | **0.0** | **10.4** | **89.6** |
| 18 | Organize a community- based study (n=145/132) | 7.5 | 59.9 | 32.7 | 0.0 | 4.5 | 95.5 |
| **19** | **Develop a public health research proposal (n=145/134)** | **6.1** | **64.6** | **29.3** | **0.0** | **6.6** | **93.4** |
| 20 | Collect research data in the field (n=142/128) | 9.7 | 58.3 | 31.9 | 0.0 | 6.2 | 93.8 |
| **21** | **Make use of information technology for effective data analysis and communication (n=143/135)** | **6.2** | **58.6** | **35.2** | **0.7** | **20.4** | **78.9** |
| 22 | Apply qualitative/ quantitative methods in public health practice (n=144/129) | 10.3 | 69.2 | 20.5 | 0.8 | 28.2 | 71.0 |
| **23** | **Write a research report (n=143/132)** | **6.9** | **64.8** | **28.3** | **0.0** | **9.0** | **91.0** |
| 24 | Apply the procedures of the Ethic committee in biomedical studies (n=142/103) | 27.1 | 57.6 | 15.3 | 1.9 | 22.9 | 75.2 |
| **25** | **Apply computer skills successfully in your work (n=144/142)** | **0.7** | **32.9** | **66.4** | **0.7** | **9.7** | **89.6** |
| 26 | Use English effectively in your work (n=143/127) | 11.7 | 66.2 | 22.1 | 8.5 | 55.0 | 36.5 |

**Table D - Leadership competencies performed by the MPH alumni**

|  | ***Skills/ competencies*** | ***How frequently do alumni use this competency in current work (%)*** | | | ***How confident do alumni feel in performing this competency (%)*** | | |
| --- | --- | --- | --- | --- | --- | --- | --- |
|  |  | *Not at all* | *Sometime* | *Very frequently* | *Not confident at all* | *Some confident* | *Confident* |
| ***Leadership competencies*** | | | | | | | |
| **27** | **Communicate with the community, mobilizing the community to participate in health care activities (n=144/136)** | **6.2** | **50.7** | **43.2** | **0.0** | **4.4** | **95.6** |
| 28 | Conduct communication activities on the community’s health problems (n=145/133) | 8.2 | 53.7 | 38.1 | 0.7 | 6.7 | 92.6 |
| **29** | **Demonstrate effective written and oral communication with the community in various contexts (n=144/139)** | **4.1** | **57.5** | **38.4** | **0.7** | **8.5** | **90.8** |
| 30 | Lobby leaders for solving community health problems (n=144/126) | 12.3 | 58.9 | 28.8 | 0.8 | 11.7 | 87.5 |
| **31** | **Create multisectoral cooperation to solve community health problems effectively (n=145/128)** | **11.6** | **59.2** | **29.3** | **0.0** | **12.3** | **87.7** |
| 32 | Facilitate group work effectively (n=145/136) | 6.1 | 54.4 | 39.5 | 0.0 | 5.1 | 94.9 |
| **33** | **Use analytical, critical thinking, and problem-solving skills to make decisions effectively (n=145/137)** | **5.4** | **55.1** | **39.5** | **0.0** | **12.2** | **87.8** |
| 34 | Work effectively within culturally diverse groups and settings (n=144/135) | 5.5 | 67.8 | 26.7 | 0.0 | 21.2 | 78.8 |
